# Observed trends in the magnitude of socioeconomic and area-based inequalities in use of caesarean section in Ethiopia: a cross-sectional study

**DOI:** 10.1186/s12889-020-09297-x

**Published:** 2020-08-11

**Authors:** Gebretsadik Shibre, Dina Idriss-Wheeler, Ghose Bishwajit, Sanni Yaya

**Affiliations:** 1 Department of Reproductive Health and Health Services Management, School of Public Health, Addis Ababa University, Addis Ababa, Ethiopia; 2 Interdisciplinary School of Health Sciences, Faculty of Health Sciences, University of Ottawa, Ottawa, Canada; 3 School of International Development and Global Studies, Faculty of Social Sciences, University of Ottawa, 120 University Private, Ottawa, ON K1N 6N5 Canada; 4 The George Institute for Global Health, The University of Oxford, Oxford, UK

**Keywords:** Caesarean section, Inequality, Global health, Ethiopia, DHS

## Abstract

**Background:**

In Ethiopia, there is a paucity of studies on inequality in caesarean section using methodologically rigorous and well-established approaches. In this study, we showed extent and the overtime dynamics of inequality in caesarean section in Ethiopia following rigorous methodologies.

**Methods:**

The data for analysis came from Ethiopia Demographic and Health Surveys (EDHS) conducted between 2000 and 2016. We used the World Health Organization’s (WHO) Health Equity Assessment Toolkit (HEAT) to analyze the data. Caesarean delivery was disaggregated by four equity stratifiers, namely education, wealth, residence and regions. Relative and absolute summary measures were calculated for each equity stratifier to capture inequality from different perspectives. 95% Uncertainty Interval was calculated around a point estimate to measure statistical significance.

**Results:**

We found large socioeconomic and area-based inequalities in use of caesarean section in all study surveys. The inequalities have occurred in favour of socioeconomically advantaged women and those living in urban areas and certain regions such as Addis Ababa. While area-related inequality had generally increased with time, socioeconomic inequality showed fluctuation. Adoption of different measures in the study for the inequality analysis has caused the emergence of mix of patterns in caesarean section inequality over time.

**Conclusions:**

In all the surveys, wealthy and more educated women, and those residing in urban areas had higher chance of obtaining caesarean delivery. Policy makers should work to ensure caesarean section that is in the accepted normal range. More emphasis should be drawn to subpopulation with under use of caesarean section while at the same time, discouraging unjustified use of it.

## Background

Caesarean section (CS) is one of the most commonly performed surgical procedures and is recognized as an important component of the essential and emergency obstetric care services [1] as it can effectively prevent both maternal and childhood mortality and morbidity [2]. Nonetheless, the number of unnecessary caesareans is rising with no evidence indicating the benefit of caesarean delivery when not medically justified [3]. The intervention carries a substantial amount of risk to the mother and infant [4, 5] and each case should be carefully considered [3, 5].

While an overall rate of a health outcome indicator such as caesarean delivery can be useful for assessing global trends, it is crucial to assess variation in CS rates for population subgroups. In the era of Sustainable Development Goals (SDGs), inequalities in access to emergency obstetric care can hinder progress towards one of the central objectives of the 2030 Agenda – that of “leaving no one behind”. In other words, even when CS utilization is at an optimal rate in a population, knowledge at the aggregate or national level has limited significance in less equitable countries because it does not signal uptake of the service by all who require it [6]. Attaining an “optimal” rate at the national level could hide the unacceptably low or high use of this service in different subpopulations in a country. Knowing the distribution of CS across the sub-populations in a country makes it possible to design interventions that target subgroups who require the service but do not have or cannot access it, while discouraging overuse among others. To understand these health inequities, it is importance to disaggregated national level data using the internationally recommended equity stratifiers for use in health equity studies [1].

Available evidence confirmed the existence of sizable inequalities in the caesarean delivery both within and between countries [7–12]. Africa has the lowest rate for caesarean section [7] among the WHO regions, and according to a study in 2016, caesarean delivery rate had not increased in Sub-Saharan Africa over the last 2 decades, when it had improved in the other regions of the world [8]. At country level, Ethiopia has one of the lowest utilizations of caesarean delivery in the world, with less than 2% of babies born by CS [7, 12]. For socioeconomic differentials in the uptake of caesarean delivery, women with higher wealth and education [7–13] were more likely to undergo CS procedures. Furthermore, women living in urban areas were more likely to access and use CS services [12] than women in rural areas, leading to an area-based disparity.

There is a dearth of studies in Ethiopia that have addressed inequality of CS among sub-groups using rigorous and well-established approaches. Existing evidence is not comprehensive, old, based on approaches [10, 11, 13] not in line with the WHO recommendation for inequality studies [14] or based mainly on disaggregation [7, 12, 13]. Current evidence based on recommended inequality analysis techniques that overcome limitations of prior studies is necessary to help close the gap between a sub-group that performs well and one that is performing poorly, thereby, facilitating the realization of the SDGs where equity is prominently featured.

The WHO recommends the use of both simple and complex, as well as absolute and relative inequality measures for inequality analysis of health care indicators [14]. The approach showcases disparities using various dimensions and measures of a health indicator to minimize reporting of ‘no inequality’ when one actually exists and vice versa. It provides a more accurate reflection of inequities among sub-populations through the use of summary measures and disaggregated analysis. Finally, using various equity stratifiers, it allows examination of disparities in a health indicator from the perspective of different population subgroups. While the reviewed literature on this topic in Ethiopia suffers from omission of one more of these important attributes of inequality analysis, our study followed all the steps which both updates and builds on the existing work.

Over the last two decades, Ethiopia has committed to creating a health care system capable of offering CS to all subpopulations who need it. It is challenging to assess the country’s pledge of reaching mothers who require CSs without evidence generated through sound methodology on the differential access to and use of CSs over time. The goal of this study was to assess the extent of and trends in socioeconomic and area-based inequalities in use of CSs in Ethiopia. Particularly, we sought to answer the following two research questions: 1) what is the extent of inequality in use of CSs in Ethiopia? and: 2) what does the time trend of both socioeconomic and area-based inequalities in use of the CS look like over the last 17 years between 2000 and 2016?

## Methods

### Study setting

Maternal death in Ethiopia is one of the highest in the world [15] and signals substantial inequality in access to maternal health care services [16, 17]. However, the country has remarkably reduced the burden of maternal and child mortalities over the last several years [15, 18]. Ethiopia had a Maternal Mortality Ratio (MMR) of 401 in 2017; MMR decreased from 298 deaths per 100,000 live births in 2000, to 401 deaths per 100,000 live births in 2017 [15]. The averted deaths may be linked to a concomitant rise in coverage of maternal and child health services interventions during the same time period [19]. However, the gains are not enough; the current maternal health care service coverage is far from the level required to achieve the SDG of 70 deaths per 100, 000 live births [20]. In 2015, Ethiopia adopted an equity-laden five-year rolling *Health Sector Transformational Plan (HSTP*) [21] to help facilitate implementation of effective interventions and ultimately meet the 2030 SDG.

### Data source

This study was performed using cross sectional data obtained from the Ethiopia Demographic and Health Survey (EDHS) conducted in 2000, 2005, 2011 and 2016. All were nationally representative household surveys conducted by the Central Statistical Agency (CSA) of Ethiopia. The EDHS is the only source of nationally representative data on several health indicators including caesarean sections in Ethiopia. Two interim EDHSs were conducted in 2014 and 2019, and both were not included in the analysis because they are not available in the HEAT software.

The DHS collects data on a wide range of health topics such as maternal health, child health, domestic violence, female genital mutilation, HIV/AIDS, maternal and child nutritional status, to mention just a few. Samples in the DHS are deemed to be representative nationally as well as regionally and for urban and rural residence. The number of women age 15 to 49 years included in the surveys were 15,683, 16,515, 14,070 and 15,367 in 2016, 2011, 2005 and 2000, respectively. The sample share of each of the waves was presented in the results section (Table 1) [14].

**Table 1:** Cesarean section rate among non-pregnant women aged 15 to 49 years disaggregated by education, economic status, place of residence and region, the EDHS between 2000 and 2016

Inequality dimensions	Category	Survey Years											
		2000			2005			2011			2016		
		Estimate	LB	UB	Estimate	LB	UB	Estimate	LB	UB	Estimate	LB	UB
Economic status	Poorest	0	0	0	0	0	0.3	0.2	0.1	0.4	0.9	0.3	2.4
	Poor	0	0	0	0.4	0.1	1.2	0.3	0.1	1.1	1.1	0.4	3.3
	Middle	0.1	0	0.5	0.2	0.1	0.7	0.5	0.2	1.3	1.6	0.8	3.3
	Rich	0.2	0	1.1	0.2	0.1	0.7	0.7	0.3	1.7	1.5	0.7	3.09
	Richest	3.4	2.4	4.9	6.1	4.6	7.9	8.3	6.3	11	9.5	7.5	11.9
	No-education	0.1	0	0.2	0.4	0.2	0.6	0.4	0.2	0.75	0.9	0.5	1.51
Education	Primary	0.6	0.2	1.5	0.9	0.4	2	2.3	1.5	3.64	3.3	2.3	4.88
	Secondary or higher	8.7	5.9	12.6	14	11	19	16	11	23.5	12	8.6	15.7
Residence	Rural	0.1	0	0.3	0.3	0.2	0.5	0.4	0.3	0.68	1.2	0.8	1.77
	Urban	5.2	3.5	7.7	11	7.7	14	9.6	7.3	12.5	13	10	16
Region	Tigray	0.3	0.1	1.31	0.7	0.2	2	3.4	2.2	5.26	2.1	1.2	3.8
	Affar	1.5	0.6	4	1	0.2	4	2.4	0.7	7.66	0.6	0.2	1.84
	Amhara	0.1	0	0.51	1	0.5	1.9	1.3	0.5	3.11	3.2	2	5.01
	Oromiya	0.5	0.3	0.91	0.7	0.4	1.2	0.5	0.2	1.08	1.4	0.8	2.26
	Somali	1.6	0.7	3.7	1	0.3	3.9	0.3	0.1	1.26	0.4	0.1	2.12
	Ben-gumz	2.9	1.4	5.8	0.3	0	1.8	2	0.9	4.51	1.3	0.5	3.11
	SNNPR	0.5	0.1	1.99	0.8	0.6	1.2	1.7	0.9	3.47	2.9	1.6	5.17
	Gambela	2.6	1.1	5.72	1.6	0.6	4.6	8.4	4.3	15.8	2.3	1	5.19
	Harari	2.7	1.5	4.6	3.9	2.6	5.8	8.6	5.9	12.2	8.8	6	12.7
	Addis Ababa	11	8	15.4	18	11	28	22	17	28.6	20	16	25.8
	Dire dawa	2.9	1.1	7.6	5.2	2.8	9.4	6	3.5	10.1	7	4.3	11.3
National rate of delivery by caesarean section (Ethiopia)^a^		0.7			1.0			1.5			1.9		

^a^ STATcompiler. The DHS Program. [Internet]. [cited 2020 Jul 13]. Available from: https://www.statcompiler.com/en/

The detailed sampling methodology of the DHS has been described elsewhere [22]. Briefly, DHS follows a stratified two stage clustered sample design. Stratification is done based on the sub-national regions and place of residence. Following stratification, samples are drawn from each stratum through a two-step process. In the first stage, census enumeration areas (EA) are selected through a sampling technique known as Probability Proportion to Size (PPS), where the probability of selection of an EAs is contingent upon its size (measured through number of households), and the larger the EA is, the higher its chance of being in the sample. Information on the number of EA is basically obtained from most recent census data. In the second stage, 25–30 households are selected systematically from each stratum.

### Study population

The surveys covered all women aged 15 to 49 years who gave birth in the 5 years preceding the survey. For the CS variable, all women who gave birth two or 3 years preceding the surveys were included.

### Measures of inequality

The inequality variable measured in this study is birth delivered through caesarean section**.** It is measured as the proportion of all births by caesarean section in the two or 3 years prior to the surveys. Restricting our analysis to two or 3 years prior to the surveys period allowed us to present a more recent status of the CS rate and its disparity. We disaggregated the caesarean birth by the four equity stratifiers: economic status, education, place of residence, and the subnational regions. The economic status (or wealth) has five categories: poorest, poor, middle, rich and richest. Educational status of the woman was classified as no-education, primary, secondary of higher; place of residence as urban vs. rural, and the sub-national regions included the nine regions and two city administrations. The disaggregated caesarean delivery (reported as a percent) was presented for each of the four EDHS time periods. Point estimates were calculated and presented with corresponding 95% Uncertainty Intervals. The educational status and wealth have a natural ordering and are known as ordered equity stratifiers whereas place of residence and regions are non-ordered equity stratifiers. Whether an equity stratifier is ordered or not affects the choice of summary measures to be calculated [14].

### Statistical analysis

We used the 2019 updated version of the WHO’s HEAT software [23] for analyzing the socioeconomic and area-based inequalities in the CS rate in Ethiopia between 2000 and 2016. The software description and access have been described in detail elsewhere [24]. In summary, the WHO released the software in 2016 using free and publicly available R programming language and R packages. The software allows assessment of within country health inequalities of more than 30 indicators for the reproductive, maternal, newborn, and child health (RMNCH). It also permits bench-marking inequality in one country with that of another country, allowing direct comparison of inequality in two or more countries at the same time [23, 24]. Since the software is publicly available, there is no ethical concern regarding access.

Type of health indicator of interest (favorable vs. adverse) and the inherent properties of dimensions of inequality determine choices and interpretations of summary measures for this inequality study. Caesarean section was disaggregated by the commonly used dimensions of inequality discussed above. In addition, we adopted summary measures of different use and statistical properties. We employed a combination of absolute and relative inequality summary measures. These were Difference (D), Population Attributable Risk (PAR), Slope Index of Inequality (SII) and Relative Concentration Index (RCI); the first three are absolute inequality measures and the last is a relative measure of inequality. These measures were calculated for each of the four equity stratifiers; for the wealth and education dimensions of inequality, we calculated all of the four inequality measures, and for the region and place of residence, we calculated the D and PAR.

The detailed methods of calculation, interpretation and all other detailed properties of the measures employed in the study have been described elsewhere in detail [23]. We offer a brief description of properties and interpretations of the measures. Whilst positive values of the measures are indicative of a disproportionately higher coverage of the service among the advantaged sub-groups (women who have completed secondary education or higher, are wealthy, are urban dwellers and live in big cities such as Addis Ababa are deemed advantaged); negative values indicate the disadvantaged nature of the service. When the absolute inequality measures are zero or the relative inequality measures are one, there is *no inequality*. The higher the absolute value, the greater the inequality is.

D is a simple measure suitable for showing the absolute difference between two categories within a dimension of inequality (i.e. urban vs rural for residence) or between 2 or more categories (i.e. regions) based on an identified reference subgroup in the category. The other three (PAR, SII, RCI) are weighted complex measures of inequality that take into account sizes of subpopulations used in the calculation, thereby producing estimates reflective of the subpopulation size [14, 23]. Complex measures are the one that take account of sizes of subpopulation used in the calculation of a measure in question, thereby producing estimates reflective of size of the subpopulation [14, 23]. The computation of SII and RCI was restricted to education and wealth dimensions of inequality since they require an ordered equity stratifier.

To declare that CS shows statistically significant disparities across the sub-groups of each equity stratifer, and to determine whether or not the inequality changed with time, we computed 95% Uncertainty Intervals (UI) around point estimates of each measure for each survey. For all inequality measures, the lower and upper bounds of the UI must not include zero to interpret that inequality exists. We assessed the trend of inequality for each summary measure by referring to the UIs for the different survey years; if the UIs did not overlap, inequality existed. The findings were presented per the recommendation of the Strengthening Reporting of Observational studies in Epidemiology (STROBE) reporting guidelines [25].

### Ethical consideration

Ethics approval is not needed as the data is retrospective, anonymous and publicly available. Ethical procedures were the responsibility of the institutions that commissioned, funded, and managed the surveys. All DHS surveys are approved by Inner City Fund (ICF) International as well as an Institutional Review Board (IRB) in the respective country to ensure that the protocols are in compliance with the U.S. Department of Health and Human Services regulations for the protection of human subjects.

## Results

### Distribution of caesarean section rate across equity stratifiers and survey years

**Fig. 1:**
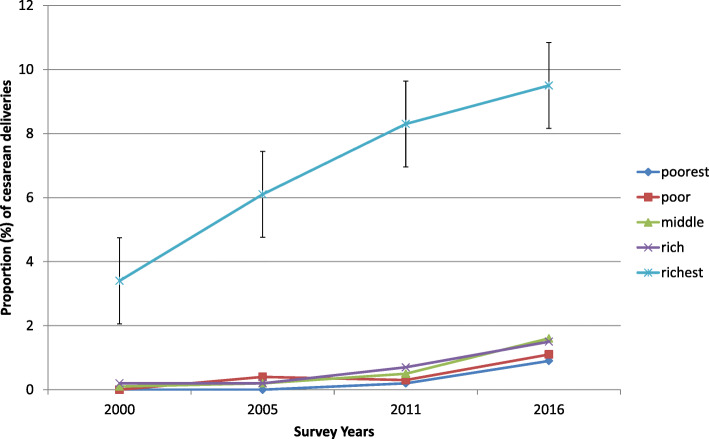
Time-trend of proportion of cesarean deliveries disaggregated by women’s economic status across the four rounds of the EDHS between 2000 and 2016

Point estimates and the corresponding 95% Uncertainty Intervals (UI) of CS rate for each of the four EDHS waves are disaggregated by the four equity stratifiers in Table 1. In 2000, the proportion of births delivered through caesarean section was zero for each of the four subpopulations of the economic status, namely the poorest, poor, middle and rich. The caesarean rate among women in the richest quintile increased from 3% in 2000 to 10% in 2016. The rate of CS has increased over time among the four bottom quintiles, but it remained below 2% in all four groups in 2016. The pace of rise among the bottom four quintiles is far slower than among the richest quintile, and this resulted in the richest fifth having much higher CS rates. The disproportionately higher utilization of the service by women in the fifth quintile seemed to have contributed to appearance and perpetuation of a considerable wealth-based inequality between the richest fifth subpopulation and the other four categories in all the survey years (Fig. 1).

**Fig. 2:**
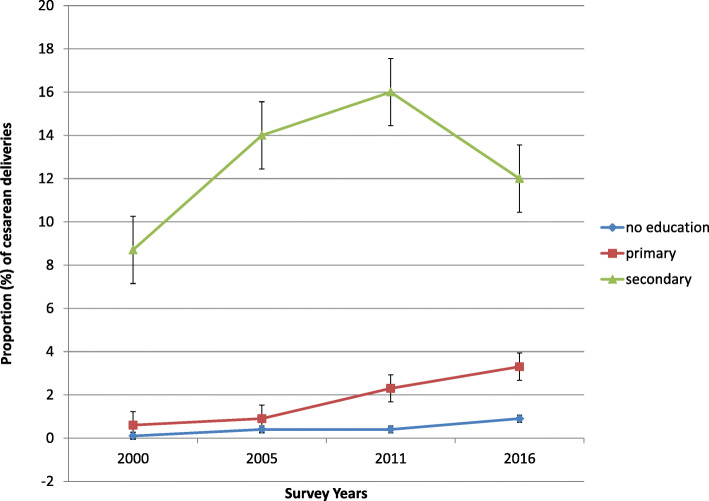
Time-trend of proportion of cesarean deliveries among women in different categories of women’s education status across the four rounds of the EDHS between 2000 and 2016

Women who completed secondary education or higher had the highest caesarean deliveries than that of women in either primary education or uneducated subgroups throughout the study period. The pace of increase in caesarean birth continued to be higher among women who finished secondary schooling than those of other sub-groups in the all surveyed years (Fig. 2). For instance, CS has risen by 3.3 percentage points (pp) among women who completed secondary education between 2000 and 2016, whereas it has increased by only 0.8 and 2.7 pp. respectively among illiterate and primary education women during the same time period. This differential rise in caesarean birth between the three categories of education status further demonstrated education-based inequality.

**Fig. 3:**
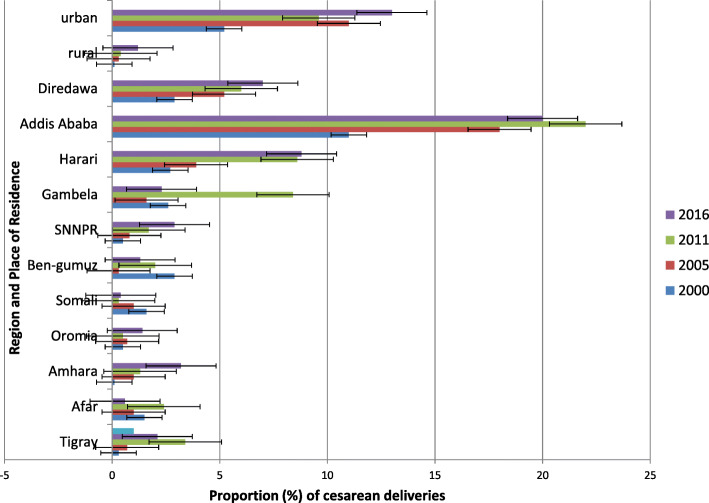
Time-trend of proportion of cesarean deliveries disaggregated by region and place of residence across the four rounds of the EDHS between 2000 and 2016

When looking at place of residence, urban dwellers and women in big cities such as Addis Ababa delivered via CS more often than their counterparts. CS among urban settings increased by approximately 7 percentage points (pp) between 2000 and 2016 compared with those in rural areas, where it improved by 1 pp. during the same time period. In 2000, no women delivered through CS, while in 2016, the Somali region had CS of only 0.4%. A larger increase in caesarean coverage among urban settings and certain regions contributed to the observed area-based inequality (Fig. 3).

### Caesarean section inequality based on different summary measures of inequality

**Fig. 4:**
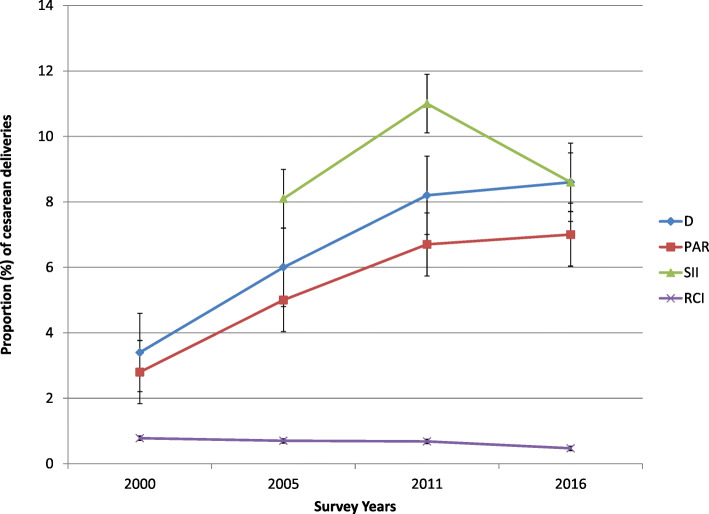
Trend of economic status related inequality in CS rate as measured by different summary measures, the EDHS between 2000 and 2016

**Fig. 5:**
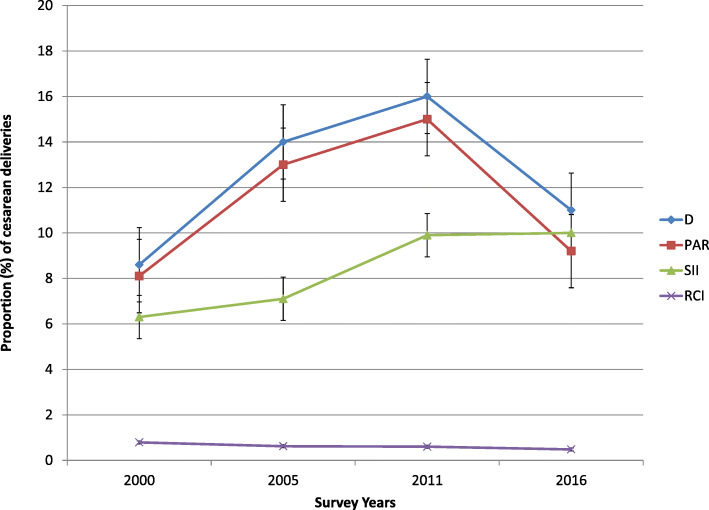
Trend of education-based inequality in CS rate as measured by different summary measures, the EDHS between 2000 and 2016

**Fig. 6:**
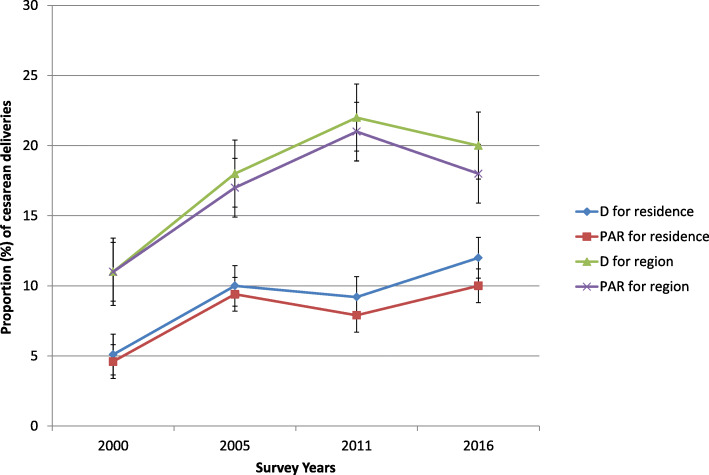
Trend of area-based inequality in CS rate between 2000 and 2016 EDHS

Table 2 presents CS birth inequalities assessed through the four summary measures and computed for each dimension of inequality of interest for our study. Economic status related inequality was observed by all the summary measures in all the studied survey years, and inequality trend varied based on the summary measures of the inequality used. Figures 4, 5 and 6 depict the line trend of caesarean delivery inequality as measured by the different summary measures of inequality. Looking at just point estimates, D and PAR showed steady rise in the wealth-based inequality, whereas SII revealed fluctuation over time. However, the wealth related inequality had steadily dropped in 17-year time based on the estimates of the RCI (Fig. 4).

**Table 2:** Inequality in cesarean section rate by different inequality measures across the various dimensions of inequality, between 2000 and 2016 EDHS

Inequality dimension		Survey years											
		2000			2005			2011			2016		
	Measures	Estimate	LB	UB	Estimate	LB	UB	Estimate	LB	UB	Estimate	LB	UB
Economic status	D	3.4	2.2	4.6	6	4.4	7.6	8.2	5.8	10	8.6	6.2	11
	PAR	2.8	NA	NA	5	4.7	5.2	6.7	6.4	7	7	6.5	7.5
	SII	NA	NA	NA	8.1	5.4	11	11	8	14	8.6	6.5	11
	RCI	78	72	84.3	70	60	81	68	60	76	47	32	61
Education	D	8.6	5.3	11.9	14	10	18	16	9.5	22	11	7.2	14
	PAR	8.1	8	8.16	13	13	13	15	14	15	9.2	9	9.4
	SII	6.3	4	8.64	7.1	4.9	9.2	9.9	7.3	12	10	7.8	12
	RCI	79	71	88	62	49	74	60	49	71	48	37	59
Place of residence	D	5.1	3.1	7.15	10	7	13	9.2	6.6	12	12	8.5	15
	PAR	4.6	4.5	4.69	9.4	9.3	9.6	7.9	7.8	8.1	10	10	10
Region	D	11	7.4	14.8	18	9.8	26	22	16	28	20	15	25
	PAR	11	10	10.7	17	−1	35	21	19	22	18	17	19

In 2000, women who completed secondary education or higher had, on average, 9% higher CS than women with no education. This difference increased to 14% in 2005 and then dropped in later years. All summary measures except RCI confirmed a marked education related caesarean birth inequality in all the survey years, with the inequality generally becoming worse overtime. The RCI estimates indicated a steady decrease across the survey years (Fig. 5). In all the studied years, the disparity has been to the advantage of the more educated women.

The study also found a noticeable area-based inequality in all the studied years. In 2000, looking at the estimates of the PAR, the national CS would have been improved by roughly 5% if rural area was performing on par with urban settings. In 2016, the potential improvement in the national CS prevalence increased to 10% if the rural area was as good as the urban area in terms of CS rates. Overall, the urban-rural inequality underwent slight fluctuation with time (Fig. 6). Similarly, the study demonstrated significant regional variations in CS rates with an evident peak in 2011.

## Discussion

This study has attempted to examine the degree and dynamics of inequality in CS over the last roughly two decades. The study confirmed the presence of a substantial amount of both socioeconomic and area-based inequalities in the utilization of the service in all the EDHS time points. Using the commonly adopted summary measures and equity stratifiers, the study confirmed the presence of both socioeconomic and area-based inequalities in the utilization of CS in all the EDHS time points.

Generally, the inequality worsened over time most likely due to a differential performance in the pace of reduction of the service among the different subpopulations; women in the higher socioeconomic gradient received a disproportionately higher percentage of the service, and had seen a higher pace of increasing the service over time compared to that of the disadvantaged subgroups. Although the national rate of CS (see Table 1) increased from 0.7 in 2000 to 1.9 in 2016 [26], the vast majority of this increase occurred in socio-economically advantaged groups and regions.

The degree of inequality varied significantly across the four EDHS across all measures, yet, wealthier woman continued to disproportionately utilize the large portion of the service. Available evidence also confirms the pro-wealthy situation of birth through CS in other low and middles income countries and specific regions most affected like sub-Saharan Africa and Asia [7–13]. For all the surveyed periods, the education related inequality in the use of caesarean section delivery had been to the favor of women who had acquired secondary education. This finding is consistent with studies carried out elsewhere [10, 13].

It was discovered that for both wealth-based and education related inequalities in the caesarean delivery, absolute inequality increased over time while the relative inequality decreased throughout the four waves. This indicates that the disparity in CS use between the socioeconomic subgroups is more pronounced using the absolute value than the gap in CS use in one group compared to the other in relative terms. For instance, RCI conclusions for education inequality contradict those of PAR and D indicating CS inequality decreased over time. Then observed variations in findings resulting from the different measures may be due to dissimilarities in statistical properties of the methods adopted in the study [14]. For instance, the PAR for wealth is calculated based on the CS rate in the richest subgroup and the national CS rate. So, both the extent of wealth variation and the national coverage of CS rate do affect the value of the PAR. The concentration index depends only on the relationship between the health variable (CS) and the national average; the rank of the living standards variable (be it wealth or education) does not affect computation of the relative concentration index [27]. Furthermore, the decrease in the relative measure and increase in absolute measure could also be due to the initial very low rate in poorest and less educated subgroup as seen in 2000 in Ethiopia. These conflicting findings between the relative and absolute measures are not uncommon; it is necessary and important to analyze them jointly to point out the different interpretations and ensure any future approaches are implemented knowing details about the findings (i.e. the whole picture) [28]. As well, each summary measure comes with different ethical underpinnings which may also partly explain the mixed conclusions regarding the inequality trends studied [14, 28]. Planners and policy makers need to understand the fundamental features of the summary measures computed in the study. That is, the use of different measures in a study prevents policy makers from creating interventions which are not adequately informed by strong evidence.

A study looking at trends in caesarean delivery by country and wealth quintile in southern Asia and sub-Saharan Africa found that caesarean delivery was predominantly concentrated among women in urban areas and in certain regions such as Addis Ababa, with the pro-urban inequality increasingly exaggerated over time [12]. The incommensurate rise in proportion of births delivered through CS over time could substantially explain the observed severe inequality, where women in higher socioeconomic positions and residing in urban settings, particularly large cities, utilize the majority of the service. The unequal distribution of this essential obstetric care, as demonstrated by the number of women in different equity stratifers, may be explained by unequal accessibility to health facilities that offer the service. CS procedures take place in acute care hospital settings with other comprehensive obstetric care services. The number of these facilities, and hence services, in Ethiopia are limited and most likely able to be accessed by economically better-off individuals.

At the same time, poorer woman in rural areas who have not completed secondary schooling underutilize the service at a level well below the optimal national level thought to inform countries about unnecessary deaths associated with obstetric complications [29, 30]. The huge variation in accessibility to important emergency obstetric care and health care resources between the different regions in the country may be contributing to the observed CS disparity in our study [31]. Standardized decomposing of the CS disparity is required to understand the determinant factors causing the disparity, and to judge whether factors were in play for the CS rate to vary between different subpopulation groups. If we do not move toward equitable access to necessary CS services, a substantial number of obstetric-caused deaths can be anticipated in the disadvantaged population. The optimal rate of CSs is thought to be 15% in a population [29]; in this study, even socioeconomically advantaged women did not use the service up to the recommended threshold, suggesting that the service is not being used beyond the required level. The exception to this generalization is for women in the capital, Addis Ababa, where coverage is well over the recommended maximum optimum value of the service (29%). The disproportionately large concentration of private and public hospitals in the city provide opportunities for women (as far as economically feasible) to give birth through CS even when it is not medically necessary. Subsequently, this scenario potentially explains the skewed distribution of caesarean delivery coverage towards urban settings [12]. Even though we see increases in CS rates among the middle, poor and poorest quintiles and women living outside major urban areas (particularly Addis Ababa) and in rural regions of Ethiopia, the rates are quite low. Government focus must be on increased access to CS with which come other challenging tasks necessary for health systems reform (i.e. skilled health workers, surgical facilities, referral systems).

The Federal Ministry of Health in Ethiopia wants to improve the maternal health status of the country and has the goal of meeting all (100%) the needs of emergency obstetric and newborn care in the country by 2025 [32]. They have created Ethiopia’s 2016 Emergency Obstetric and Newborn Care (EmONC) Assessment Report, a national census of all facilities that provided delivery services, to inform policy makers, planners, researchers, and program managers [33]. The report highlighted that only 18% of all Ethiopia’s health facilities provided treatment for obstetric complications, there were gaps in necessary human resources and training to perform emergency obstetric care, and 73% of women with obstetric complications admitted to health centers had to be referred to higher level care elsewhere [33]. As we talk about the importance of access to caesareans for particular subgroups, it is important to also consider the other issues related to provision of this type of care. In 2016, Ethiopia only had 40% of the recommended number of fully functioning EmONC facilities. Furthermore, only 14% of expected deliveries took place in a functioning EmONC meaning most birth sites were not able to adequately treat obstetric emergencies [33]. Regional differences in referral systems, number of quality assurance officers, availability of water source and electrical power are some of the challenges [33]. Many of these issues must be considered as policy makers address regional disparities in the provision of emergency obstetric care [34, 35].

The study has several strengths. First, the adoption of several measures of inequality which contributes to the quality of evidence contained in this paper. Using both relative and absolute inequality measures in the same study has the potential to help investigate the magnitude and trend of inequality from various dimensions and perspectives. Secondly, the study presented the inequality findings for each subgroup of the equity stratifiers, and this can assist the government to identify where and how to focus their efforts towards realization of the equity-oriented SDG in relation with maternal health [36]. Specifically, the government needs to draw increased attention to women who are poor, uneducated, live in rural areas and in most regions to increase reasonable rates of the service. Increasing coverage of at least secondary education, increasing different financial schemes to alleviate poverty and increasing media coverage about the importance of the service could help to minimize the disparity as well as reach those who require the emergency service. Finally, the study used the high-quality data available through the WHO health equity monitor database which strengthens the quality of the conclusions drawn from the study. However, the study has some limitations. It focused entirely on description of the nature of caesarean delivery inequality in light of the recommended dimensions of health inequality [34, 35]. An in-depth assessment of impact of potential determinant factors that underlie the observed inequality requires a decomposition technique. Future studies need to apply this statistical method to better appreciate the contributions each determinant factor brings about to the observed CS disparity. Also, the study presented only the population level inequality of the service, and studies are required to see whether the same disparity remains at health facility level. Although our findings may be particular to Ethiopia’s context, we believe there are lessons learned which may be helpful to other countries with large areas and potentially similar disparities.

## Conclusions

Findings from this study illustrate the continued inequality in caesarean delivery to the advantage of women in higher socioeconomic positions who reside in urban settings, with the inequality intensifying over the time period of the study. Despite increases in national caesarean rates and those among socio-economically disadvantaged women, caesarean rates remain critically low (below 5%) suggesting that these women are suffering the morbidity and mortality consequences of a lack of access to caesareans.

Policy makers need to target subgroups which are performing poorly compared to both the minimum recommended level and those who are using the service well. Without formulating a strategy that targets those subgroups (subpopulations at the lower end of the socioeconomic spectrum and those in rural areas), it is unlikely the country will avert deaths associated with unforeseen obstetric emergencies. Potential deaths of both mothers and newborns could be stopped if women who require a caesarean section are able to access one. Lack of strategic planning hinders achievement of the maternal mortality related SDG. Further, these strategies need to also address unjustified use of the caesarean delivery by some subgroups because it poses substantial risk, that could be avoided, to both the mother and her child.

## Data Availability

This study was performed using cross sectional data obtained from the Ethiopia Demographic and Health Survey (EDHS) for the periods of 2000, 2005, 2011 and 2016 available here: https://dhsprogram.com/What-We-Do/survey-search.cfm?pgtype=main&SrvyTp=country

## Abbreviations

EDHS: Ethiopia Demographic and Health Survey (EDHS)
WHO: World Health Organization
CS: Caesarean section
HEAT: Health Equity Assessment
NCDs: Non-Communicable Diseases
D: Difference
PAR: Population Attributable Risk
SII: Slope Index of Inequality
RCI: Relative Concentration Index
